# Analysis of the causes, psychological mechanisms, and coping strategies of short video addiction in China

**DOI:** 10.3389/fpsyg.2024.1391204

**Published:** 2024-08-06

**Authors:** Mingyue Liao

**Affiliations:** Business School, Southwest University of Political Science & Law, Chongqing, China

**Keywords:** short video addiction, psychological mechanisms, algorithms, algorithmic loop, government supervision

## Abstract

Short video addiction refers to the uncontrollable desire of users to watch short videos, leading to significant behavioral loss of control or attention disorders, which in turn result in difficulties in social interaction, learning, and work adaptation. With the “invasion” of short videos into people’s daily lives and their spread among underage groups, the issue of short video addiction has attracted widespread social attention. Firstly, based on the causes of short video addiction, this study analyzes it from four levels: algorithm design, content services, platform control, and user experience. Secondly, combining relevant scientific theories, the psychological mechanisms of short video addiction are explained from four levels: cognition, emotion, motivation, and social factors. Finally, in terms of coping strategies, on the theoretical level, further research on the occurrence mechanism of short video addiction should be deepened, and attention should be paid to the influence of recommendation algorithms on short video addiction. On the practical level, the obligations and responsibilities of relevant stakeholders such as short video producers, platforms, and regulators in preventing short video addiction should be clarified, aiming to promote prevention and management of short video addiction.

## 1 Introduction

**Figure 1:**
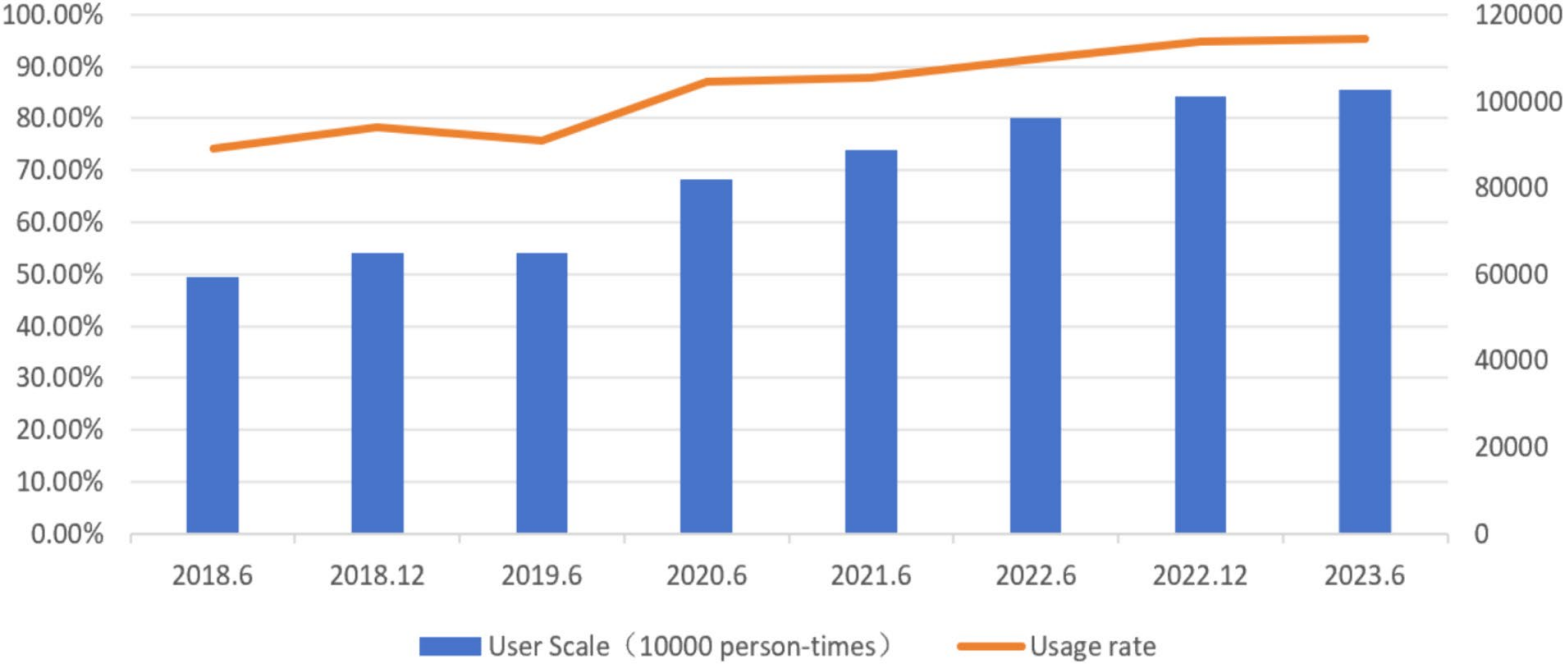
Scale and usage rate of short video users from June 2018 to June 2023. Data Source: Statistical Survey on Internet Development in China.

With the rapid development of digital technology, people’s lifestyles have undergone revolutionary changes, giving rise to a new form of social media – short videos. Short videos refer to online videos with durations ranging from a few seconds to a few minutes, which are published and shared through social media platforms, video-sharing websites, mobile applications, and more. Short video apps such as Douyin (TikTok), Kuaishou, and Tencent Weishi have formed a “individual-short video-society” structure in the current social relationship chain. Due to their rich content, concise interaction, light-hearted humor, and wide age appeal, short videos have gained popularity among a large number of users. The phrase “Five minutes on Douyin, one hour in the real world” humorously captures the empathetic feeling of people getting immersed in short videos without realizing it. According to the 52nd Statistical Report on Internet Development in China published by the China Internet Network Information Center, as of June 2023, the number of short video users reached 1.026 billion, with a user penetration rate of 95.2% (China Internet Network Information Center, 2023a). Nearly one-fourth of new internet users were introduced to the internet through short videos, and the average daily viewing time of short videos exceeded 2.5 h per person (Media Review, 2023). The 5th National Survey on Internet Use by Minors shows that the proportion of underage internet users who frequently watch short videos has increased from 40.5% in 2018 to 54.1% in 2022, making it an important channel for minors to obtain information (see Figure 1; China Internet Network Information Center, 2023b). Hormonal changes caused by brain growth and development can affect the self-control of minors and expose them to higher risks of mental health issues (Casey, 2013). Due to the “invasion” of short videos into users’ daily lives and their spread among underage groups, short video addiction has led to numerous negative incidents, severely impacting the healthy development of addicts and even disrupting normal social order.

As a result, the issue of short video addiction has gradually attracted the attention and even resistance of the public.

Critics have compared short videos to electronic fentanyl, electronic opium, and electronic cocaine, elaborate slot machines and a potential catalyst for a new era of opium wars. From their perspective, short videos are perceived as a relentless time sink, and individuals find themselves unable to break free from its addictive grip. In recent years, there has been a growing body of research on the addiction to short videos. In related research, short videos are often considered a form of network social media, and their unique impact on users has not been given sufficient attention. As network social services continue to diversify, various online platforms offer different functions, cater to distinct user groups, and operate under varying social modes (Cinelli et al., 2021). Leung and Chen (2021) proposed in their literature review that future research on Internet addiction should concentrate on specific behaviors or types of content. Some scholars also advocate for paying attention to the distinctions between short videos and traditional social services in order to thoroughly investigate their impact on users (Masciantonio et al., 2021). In addition, in terms of research subjects, scholars have conducted cross-group studies on various populations, including adolescents (He and Zhou, 2021), college students (Wang, 2021), elderly individuals (Jia et al., 2023), and rural left-behind children (Liang, 2023). On the whole, minors have received the most attention, and researchers tend to attribute the causes of addiction to intrinsic psychological states, media literacy, and external factors such as social and family structures, as well as the content and usage of short videos (Meng and Leung, 2021). In terms of research methods, researchers often employ empirical methods to explore the mediating effects of specific factors such as social-technical factors (Xie and Du, 2023). This paper focuses on short videos as a medium of information. Firstly, based on a review of relevant literature and the characteristics of internet addiction and short videos, the concept of short video addiction is defined. Secondly, the causes of short video addiction are explored from four levels: algorithm design, content services, platform control, and user experience. Finally, drawing on relevant psychological theories, the psychological mechanisms of short video addiction are deeply explored from four levels: cognition, emotion, motivation, and social factors, aiming to provide a systematic understanding of this issue.

## 2 Conceptual definition of short video addiction

There is no consensus in the academic community regarding the definition of short video addiction. “Addiction” stems from the term “dependence” and refers to uncontrollable usage behavior of social media, which is often considered to have addictive tendencies (Ryan et al., 2014). In fact, phenomena such as internet addiction and gaming addiction have long existed and have gradually expanded from the media field to the realm of mental health. The term “internet addiction” was first proposed by American psychiatrist Ivan Goldberg, but there is still debate as to whether internet addiction is a disease or a behavioral dependency. In China, the Ministry of Health denied the classification of “internet addiction” as a clinical diagnosis in 2009 (Southern Metropolis Daily, 2009). Currently, there is no specific research indicating the symptoms or clinical diagnostic criteria for short video addiction. To standardize terminology and reduce conceptual confusion, this article chooses the relatively neutral term “addiction.” In 2018, the National Health Commission of China released the “Core Information and Definitions of Health Education for Chinese Adolescents,” which defines addiction as “the impulsive behavior of internet use without the influence of addictive substances (China Health Education Center, 2018).” Undoubtedly, the usage patterns of short video platforms play a significant role in the formation and maintenance of short video addiction. Short video addiction refers to a chronic or cyclical state of obsession caused by repeated use of short video apps such as Douyin (TikTok), accompanied by intense and persistent cravings and a sense of dependence (Li et al., 2021). Some studies have focused on short video addiction but did not provide specific definitions (Nong et al., 2023). Take Instagram addiction as an example. In recent years, there have been numerous relevant studies, including the use of addiction as moderator to investigate the relationship between Instagram overuse and stress and emotional fatigue (Sanz-Blas et al., 2019). Other factors were utilized as mediating variables to examine the drivers and outcomes of Instagram addiction (Ponnusamy et al., 2020), and the relationship between Instagram addiction and personality (Kircaburun and Griffith, 2018). None of these studies have provided a clear definition of addiction to short videos, but these controversies do not hinder the continuous progress of related research and even serve as a driving force for scholars in the field to conduct further studies. The field of short video research is no exception, and the controversy surrounding the concept may persist for some time, but it does not impede the gradual emergence of research on short video addiction. In fact, existing studies on short video addiction almost entirely draw on concepts from related fields such as internet addiction, lacking unique considerations for short video addiction behavior (Zhang et al., 2019). The ambiguity of the concept indicates that current research on short video addiction is still in its early stages.

The article argues that defining the concept of short video addiction requires a combination of research on “internet addiction” and the unique characteristics of “short videos” themselves. On one hand, it is necessary to revisit the exploration of “internet addiction.” In 1980, pathological gambling was included in the Diagnostic and Statistical Manual of Mental Disorders (DSM-III), opening the theoretical possibility for the study of non-substance addictions such as internet addiction (Griffiths, 2000). Subsequently, academic attention on internet addiction has increased. In 2014, a study compared different diagnostic criteria for internet addiction, such as the components model and the Internet Addiction Test and identified three common features: (1) lack of control over internet use, (2) resulting psychological, social, or occupational conflicts or problems, and (3) mental distress (van Rooij and Prause, 2014). Furthermore, they called for attention to specific addictive behaviors. On the other hand, it is important to enhance the recognition of “short video characteristics.” The concept of internet addiction is too broad and vague for various specific forms of inappropriate internet use. Defining short video addiction requires focusing on its unique characteristics. Given that each short video platform has a characteristic structure, unique features, varied use habits, and different gratifications and motives underlying its use, it is necessary to investigate each platform addiction alongside potentially related factors. However, this paper focuses solely on the overall level of research into short video addiction and does not take into account the specific factors of each individual platform. In terms of content, short videos share the general characteristic of internet content—abundance. The vast amount of short video content makes it more appealing to users. In terms of form, short videos have distinct features: (1) short duration and fast-paced, (2) rich and varied audiovisual symbols, and (3) strong social nature, as short videos were inherently endowed with social attributes from their development (He and Zhou, 2021). Based on these considerations, this article proposes a descriptive concept of short video addiction by combining the three features of internet addiction and the characteristics of short video content and form: short video addiction refers to a user’s uncontrollable desire to watch short videos, leading to evident behavioral loss of control or attention disorders, resulting in difficulties in interpersonal relationships, learning, work, and other aspects of adaptation. The most tangible aspect of addiction can be reflected in the duration or frequency of daily short video consumption, but the deeper level of “uncontrollable desire” requires careful examination of individual users. For example, being deeply immersed while watching short videos or experiencing feelings of loss or sadness when not watching them for an extended period. It is necessary to clarify that this descriptive concept aims to promote academic attention and research on short video addiction and does not have diagnostic significance.

## 3 Analysis of the causes of short video addiction

Short video addiction is a possible outcome of user interaction with short video services. Design ethicist Tristan Harris believes that the problem of user addiction is not due to a lack of willpower but rather because thousands of people behind the screen are working hard to undermine your self-control (Alter, 2018). Short video platforms manipulate users subtly through the artistic treatment of information dissemination technology and audiovisual product design. The increase in user addiction, user stickiness, and behavioral data are important capital for such applications. This article will analyze the causes of short video addiction from four aspects: algorithm design, content services, platform control, and user experience.

### 3.1 Algorithm design

One of the main reasons for the rapid popularity of short videos is the algorithm’s “feed.” Personalized algorithms can collect users’ interests, preferences, and behavioral records, and accurately push short videos to users’ mobile devices using various techniques, such as recommendation techniques based on previously selected content or friend relationships (Geschke et al., 2019). Personalized algorithms represent a significant change for social media because they free people from a large amount of irrelevant information, allowing users to be entertained based on algorithm-recommended content without the need for active searching or selection.

In China, the early application of algorithmic recommendation technology was seen in the “Toutiao” app launched in 2012. At that time, the news field was fiercely competitive among the four major portal websites: Sina, Sohu, Netease, and Tencent. Within 3 months of its launch, “Toutiao” gained 10 million users and quickly disrupted the landscape of internet news and information. The subsequent “Douyin” app successfully replicated the algorithmic recommendation technology. It can be said that the platform, utilizing artificial intelligence and big data technology through algorithm models, tracks users’ reading preferences, matches user behavioral data with content information data, achieves highly aggregated content, and delivers it precisely. The application of algorithms has transformed the process from “people searching for information” to “information finding people,” rapidly iterating recommended content in seconds, and dynamically adapting to users’ moods and interests.

**Figure 2:**
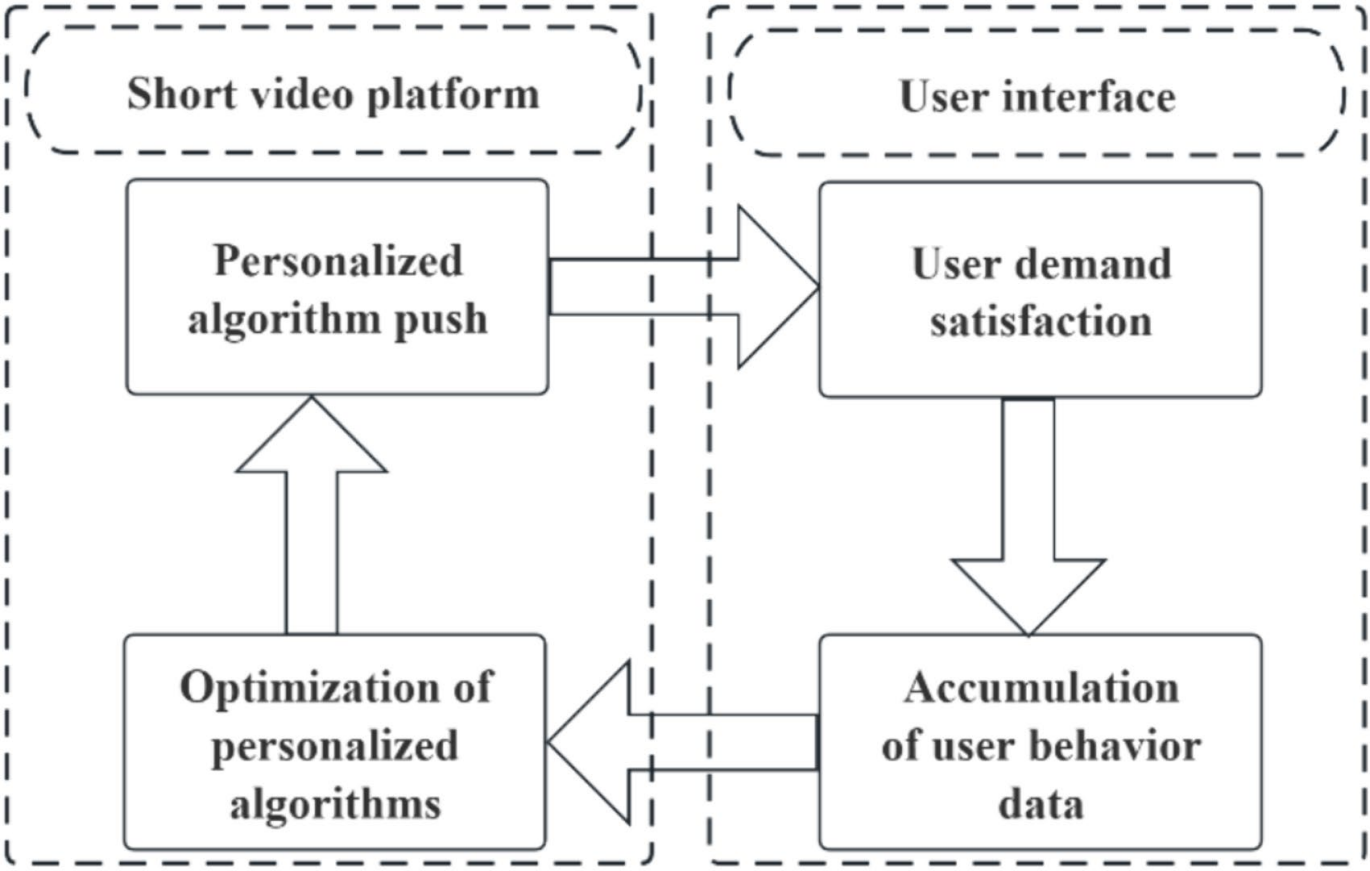
Algorithm “closed loop”.

This model significantly reduces the cost of accessing information and changes people’s habits and experiences of obtaining information on the internet. With the accumulation of user data, algorithms can continue to optimize and refine the information delivery mechanism, making it more personalized and precise (Zhang and Liu, 2021). Following the cycle of “personalized algorithm push – user demand satisfaction – accumulation of user behavioral data – optimization of personalized algorithms – personalized algorithm push – …” (as shown in Figure 2), users may become unable to make independent choices and become immersed in this effortless and enjoyable way of receiving information, unaware of the invisible control behind the scenes, ultimately leading to short video addiction or even addiction (Qin et al., 2022).

Algorithms, as neutral tools, are inherently neither good nor evil. However, the algorithm models constructed by commercial companies are imbued with a profit-oriented value system, essentially being “pseudo-neutral” technologies filled with commercial logic (Li, 2018). Research has also shown that watching personalized recommended short videos continuously stimulates the ventral tegmental area (VTA) of the brain, which is the neural circuit responsible for pleasure and reinforcement motivation. Prolonged activation of this area can lead to cravings and addiction in individuals (Su et al., 2021). It can be said that short video apps utilize AI to provide targeted recommendations based on big data and individual browsing habits. The longer the usage time, the clearer the user’s profile becomes. Users repeatedly reinforce their usage habits through the algorithm’s “precise feeding,” leading to a state of addiction.

### 3.2 Content services

Short videos are brief online videos typically ranging from a few seconds to a few minutes in length. The compression of video duration increases the accessibility of information entertainment for people living in a fast-paced society, and as a result, short videos have gained widespread user traffic. Through case studies, scholars have found that short videos better align with people’s busy lifestyles as they can provide sufficient rich information stimulation within limited time (Zea and Jung, 2019). Research has also found that the fragmented dissemination mode of short videos perfectly meets users’ need for mental pleasure during fragmented time (Qin et al., 2022).

Although most short videos are only a few seconds to a few tens of seconds long, they contain sufficient elements. This richness can be seen in two aspects: presentation style and content of information. Taking Douyin as an example, in terms of presentation style, short videos are typically composed of lively melodies, eye-catching text, and corresponding video clips. This “multichannel” sensory stimulation enhances users’ experiential sense of information content, further reinforcing their short video usage behavior. Research has shown that when users interact with a system, pleasant or stress-relieving experiences encourage them to repeat such interactive behavior (Liu and Chang, 2016), and the same applies to using short videos. The rich stimuli in short videos activate users’ sense of pleasure, alleviate negative emotions, and lead to users increasing their frequency and duration of use to reinforce this behavior.

In terms of information content, the Douyin short video platform is highly diverse, covering various themes such as creative humor, music and dance, life sharing, children’s education, emotional stories, fashion and beauty, talent skills, news highlights, dramatic performances, food Mukbang, and sports and fitness. The greater the variety, the more it can attract people with different preferences to join Douyin and meet the information needs of different users. For example, emotional story short videos are short films with emotions as the main theme, usually including romantic, touching, and inspirational content. These types of short videos are suitable for young female users and those who enjoy a romantic and artistic atmosphere (as shown in Table 1). If the information content is further divided, short videos can be categorized into functional and hedonic types. Functional short videos typically feature news, policies, knowledge and other informative content, aiming to provide users with practical efficiency and meet their functional needs. On the other hand, hedonic short videos usually revolve around humorous or cute pets, appearances, song and dance performances as their main content, offering users hedonic pleasure and meeting their entertainment needs. Some scholars have conducted research to explore the relationship between two types of videos and users’ usage behavior. It has been observed that the continuous use of functional short videos by users is inadequate, while hedonic short videos captivate a large number of users due to their appeal in terms of interest, novelty, stimulation and aesthetics. This phenomenon has led to significant social consequences (Tian, 2023). Therefore, the focus of this paper is mainly on the issue of addiction to hedonic short videos.

**Table 1:** Information content of Douyin short videos and user groups.

Short video information content	Video elements	User groups
Creative humor	Humorous plots, performance art, parody	Young users, active internet users
Music and dance	Popular music, choreography, participation of celebrities and internet celebrities	College students, female users, entertainment enthusiasts
Life sharing	Daily life recordings, practical tips, travel guides	Diverse interest users, knowledge-seeking audience, travel enthusiasts
Children’s education	Children’s teaching, animations, practical applications, storytelling	Children users, parents, educators
Emotional stories	Emotional rendering, character development, realistic scenes	Female users, urban office workers, users who enjoy romantic and artistic atmosphere
Fashion and beauty	Product showcases and demonstrations, trend interpretation	Young women, users in emerging markets
Talent and skills	Performance showcases, interactive challenges, teaching guidance	Professionals, homemakers, art enthusiasts
News highlights	Professionals, homemakers, art enthusiasts	Male users, office workers, industry professionals, elderly population
dramatic performances	Script creativity, scene arrangement, editing techniques	Users with emotional appeal, drama enthusiasts, consumers of fragmented time
Food Mukbang	Food exploration, recipe tutorials, food Mukbang	Young users, food enthusiasts, family audiences
Sports and fitness	Demonstration of exercises, equipment usage methods, dietary advice and nutrition guidance, fitness progress showcases	White-collar office workers, fitness enthusiasts, professional athletes, and coaches

Additionally, Douyin was officially launched in China in September 2016, marking the beginning of the explosive growth of short videos. However, due to its relatively brief history, the lack of government regulation has created a “lawless land” for short videos. This has led to the proliferation of low-brow, borderline violent, bloody, wealth-worshipping, and gluttonous content with misguided value orientation and bad guidance easily accessible. As a result, children may be curious about these videos and thus subjected to significant psychological impact. Furthermore, even adults may succumb to addiction to these negative videos under the pressures of work and life.

### 3.3 Platform control level

The concise human-computer interaction mode and rich content information experience are the advantages and characteristics of short videos (Tian et al., 2023). The attribution of addiction cannot be limited to the tension of audiovisual art in short videos, the lack of self-control in users, and social environmental factors, while ignoring the platform entity (Zhang and Liang, 2023). In theory, the platform economy is characterized by “double supervision,” where effective oversight can help mitigate the risk of users becoming addicted to short videos. Firstly, the government supervises platforms and merchants through the implementation of laws and regulations. Secondly, platforms themselves regulate merchants and users through access qualification reviews, monitoring transaction behavior, as well as accumulating behavior and credit data. However, the absence of government oversight has somewhat encouraged platforms to prioritize economic gain to a certain extent. This profit-driven mindset tends to undermine the regulatory willingness of platforms and may even actively entice users, especially teenagers, into excessive indulgence. In the commercial logic of the platform entity, inducing user addiction is to further obtain user labor and promote the development and growth of the platform by leveraging the creativity of the masses. Because internet technology activates individuals as information nodes, the idle resources of the masses become an important content treasure trove for the platform, providing an inexhaustible source of creation (Yu et al., 2015). In fact, platforms can induce user addiction through methods such as full-screen design and time perception interference.

In terms of page design, short video apps predominantly feature full-screen playback mode, which serves two purposes: firstly, users do not need to make choices during usage, so they are not interrupted during the process and can immerse themselves in the content pushed by the algorithm, obtaining a sense of satisfaction and joy; secondly, the visually impactful and immersive nature of full-screen design makes it difficult for people to anticipate the content of the next video, further stimulating their desire to constantly chase after information. However, long-term exposure to highly accessible stimuli can potentially lead to addiction risks, as evidenced by research in the field of addiction. For example, the high accessibility of stimuli increases the risk of addiction (Volkow et al., 2019). The portability, immediacy, and ease of use of mobile electronic games can lead to more severe gaming addiction risks (Wang et al., 2019).

In terms of time perception, short videos, due to their varying and fragmented durations, increase the difficulty for users to perceive time. The accumulation of fragmented time results in a longer passage of time. It can be said that elements such as full-screen playback, algorithmic recommendations, and fragmented lengths are all intended to disrupt the concept of time and interfere with users’ estimation of time. Over time, the experience of constantly encountering freshness and quickly accessing core content within a minute makes it difficult for users to accept long videos. This altered viewing behavior, designed by the platform, is continuously reinforced as a form of dependence, weakening users’ critical thinking, and even causing restlessness and emptiness. It is evident that short video platforms put considerable effort into disrupting users’ perception of time as much as possible.

Media scholar Shrum proposed a formula to explain people’s choices of different media: Probability of choice = Potential rewards/Effort required (Wilbur, 1984). In other words, when the potential rewards from using a particular medium are greater while the effort required is lower, the probability of users choosing that channel will be higher. For short video users, all they need to do is “swipe up” to access a wealth of information flow, significantly reducing the cost of obtaining information satisfaction. If we consider cognitive investment as the cost and the obtained stimulus satisfaction as the benefit, compared to activities that require higher cognitive participation, such as playing games, or activities that require long periods of focused attention, such as watching movies, the use of short videos is clearly a “low investment, high return” resource exchange activity. This high profitability will continuously reinforce people’s use of short videos, thereby triggering addiction risks.

### 3.4 User experience level

Previous research on internet addiction has shown that the sense of immersion individuals experience during use is one of the key driving factors of addiction (Seo and Ray, 2019). Immersion leads individuals to experience a high sense of control, heightened awareness, and focused attention, while disregarding their surrounding environment and the passage of time (Michailidis et al., 2018). When users interact with short videos, which are “actively recommended, information-rich, and interactively simple” media, they tend to focus more on the current stimuli and pay less attention to the past or future. This is known as “immersion.” Short videos can create a sense of immersion for users through first-person perspective content, and when individuals watch algorithmically recommended short videos on personal accounts, the brain’s cognitive control regions are inhibited, making it easy to enter an immersive state (Su et al., 2021). This low-cost immersion can pose significant addiction risks for short video users.

It should be noted that the flow theory, which is like immersion, is often used to explain the reasons why individuals experience immersion. In fact, many scholars have used the concept of mental flow to explain the internal mechanisms of addiction in studies on internet addiction or gaming addiction (Chou and Ting, 2003; Liu and Chang, 2016). However, the state of addiction to short videos may not be the same. Flow and immersion have certain differences, and this distinction is a key point in differentiating short video addiction from general internet addiction. Specifically, flow refers to a holistic sense of complete engagement in an activity, representing an optimal experience (Leung, 2020). This state includes high levels of competence and control, high levels of challenge and arousal, focused attention, internal enjoyment, and a sense of purpose and achievement. Therefore, one condition for achieving flow is to strike a balance between an individual’s abilities and the challenge level of the task (Yang et al., 2014; Liu and Chang, 2016). When using short videos, users can obtain rich information stimuli with low interaction costs, which does not meet the prerequisites for a flow experience. In fact, immersion leans more toward a “sense of presence,” a subjective experience that allows the subject to generate certain associations and have a strong sense of involvement. Flow, on the other hand, emphasizes a state of “total concentration” that requires strong operational skills. Furthermore, in terms of perceptual and cognitive experiences, immersion leans more toward perceptual experiences, where sensory stimuli prompt users to immerse themselves more in the current environment. Flow, on the other hand, leans more toward cognitive experiences, manifested as a mental state resulting from strong cognitive behaviors after receiving timely feedback. In summary, although immersion and flow have slight structural differences, they represent different psychological phenomena (Michailidis et al., 2018). Immersion is not an extreme state and does not require a high level of interactive balance or perceptual enjoyment. Compared to flow, immersion may be more suitable for describing the focused state of individuals when using short videos.

It is important to note that while technology plays a role in promoting addiction to short videos, it would be incorrect to solely attribute the problem to technical factors. Not all users become addicted due to algorithm technology, and whether a user becomes addicted to short videos is closely related to their own psychological factors. From an experiential perspective, the inability of users to satisfy their psychological needs offline is a significant contributing factor to their addiction to short videos. On one hand, psychologist Carl Jung introduced the concept of the inner child, proposing that within each individual resides a vulnerable, wounded, and dependent child in need of nurturing care. The reason why many users are addicted to short videos, or even unable to extricate themselves, is because it invisibly meets the psychological needs of their “inner children.” Wonderful short videos not only create a virtual entertainment space but also provide a variety of emotional values for the audience, satisfying their spiritual needs that are desired but not available offline. On the other hand, according to Maslow’s hierarchy of needs theory, needs are categorized into five levels: physiological needs, safety needs, love and belongingness, esteem and self-actualization. With the advancement of the social economy, people’s basic needs have been largely satisfied, leading to a growing emphasis on spiritual fulfillment such as a sense of belonging, respect, and self-actualization. The rapid growth of short videos caters to the higher-level demands of people. In comparison to the tightly connected social patterns in real-life society, short videos establish a more gentle and convenient pattern of interpersonal interaction. This social model of weak association caters to the tendency of current users, particularly young users, to seek escape from strong interpersonal relationships in real life. Additionally, it facilitates the identification of similar groups and the development of self-identity.

## 4 Psychological mechanisms of short video addiction

To gain a deeper understanding of the psychological mechanisms behind short video addiction, it is necessary to analyze it from a scientific theoretical perspective. The following sections will provide theoretical explanations of short video addiction from cognitive, emotional, motivational, and social dimensions, aiming to enhance public awareness of short video use and provide a scientific basis for research and intervention regarding short video addiction.

### 4.1 Cognitive dimension

In the cognitive dimension, the Dual Process Theory can be used to explain short video addiction. The Dual Process Theory, proposed by psychologists Shiffrin and Schneider (1977), suggests that cognitive processing consists of two systems: “automatic processing” and “controlled processing.” “Automatic processing” refers to well-practiced behavioral patterns, where specific steps and instructions have become nearly “unconscious” actions. “Controlled processing,” on the other hand, is limited by cognitive resources and requires conscious attention and adjustment based on the environment. According to the Dual Process Theory, due to limited cognitive resources, when the controlled processing system is weak or impaired, the influence of the “automatic processing” system on behavior is greater. Conversely, when the “controlled processing” system is strong or intact, the influence of the “automatic processing” system is smaller. To make rational and effective decisions to achieve goals, individuals must ensure that the controlled processing system can exert its supervisory and control functions to suppress impulsive behavior driven by the automatic processing system (Saunders and Over, 2009).

In recent years, the Dual Process Theory has been frequently used to explain the internal mechanisms of addiction. Studies have found that the formation of addictive behaviors is related to the enhancement of the “automatic processing” system and the weakening of the “controlled processing” system (Yan et al., 2021), which is supported by neurophysiological research (Koob and Volkow, 2010). Another study showed that during the process of watching personalized short videos, the default mode network (DMN) in the brain was activated and coupled with visual and auditory pathways but coupled less with the prefrontal cortex and cingulate cortex. This indicates that attentional resources are highly focused on visual and auditory information processing, while difficulties arise in attention regulation. At the same time, regions involved in cognitive control are inhibited, which may lead to a loss of control over short video use (Su et al., 2021). This study reveals the cognitive processing patterns of the brain during short video use from a neurobiological perspective. Based on this, due to the low cognitive engagement when interacting with short video media, the “automatic processing” system continues to be reinforced while the “controlled processing” system is restricted. As a result, even if the individual is aware that their behavior deviates from the overall goal, they may struggle to resist the impulse to repeat the behavior, ultimately leading to short video addiction.

### 4.2 Emotional dimension

In the emotional dimension, the Opponent Process Theory can be used to explain users’ addiction to short videos. The Opponent Process Theory originally explains how we perceive colors through opposing neural processes. It suggests that certain colors are linked, forming pairs that inhibit each other. When one color in a pair is stimulated, the other is suppressed, creating a balance in our visual experience. Emotions are reactions within the human inner world and play an important role in people’s daily lives. Emotions arise from individuals’ evaluations of things, where positive evaluations lead to positive emotions and attraction toward the object, while negative evaluations lead to negative emotions and avoidance of the object (Strongman, 2006). Emotions have a peculiar characteristic that when a strong emotion subsides, it is often naturally accompanied by an opposite emotion. Opponent process theory is an explanation of how the experiences of certain sensory and neurological phenomena are linked together. Put simply, the body efficiently processes opposing experiences, such as fear and pleasure, at the same site, making it difficult for people to experience both at once. When stimulation at such a site evokes one experience, a person may experience an “afterimage” of the opposite experience after the stimulation is over. The Opponent Process Theory has been widely used to study addiction, child attachment, and sensation-seeking behaviors (Lee et al., 2014). The theory describes the underlying positive reinforcement and negative reinforcement mechanisms and intuitively explains why specific systems can lead to addiction through the opposing processes of positive and negative emotions (Tian et al., 2023).

Research has shown that the stimulation from short videos activates the brain’s reward pathway. The cerebellum, as part of the reward pathway, is directly connected to the ventral tegmental area, which is one of the core regions for processing rewards (Carta et al., 2019). Personalized short videos, compared to regular short videos, more significantly activate the ventral tegmental area because the algorithm-recommended videos have higher reward value for users and can produce a greater pleasure effect. As a relaxed and enjoyable form of entertainment, short videos provide users with a temporary escape from busy work or study. In other words, watching short videos has a pleasurable effect. At this point, users’ positive emotions are activated. However, in real-life situations, due to psychological or external factors, users often must stop or temporarily pause their use of short videos. Once the stimulation stops or decreases, positive emotions are lost, triggering negative emotions such as anger and stress. To maintain positive emotions and reduce negative emotions, users must repeatedly activate the behavior of using short videos to maintain the sense of pleasure, ultimately leading to short video addiction (Tian et al., 2023). Therefore, systematically discussing the psychological needs of short video users under the Opponent Process Theory, clarifying the usage mechanisms driven by different emotions, can provide scientific guidance for the rational use and management of short videos.

### 4.3 Motivational dimension

In the motivational dimension, the Social Shaping of Technology theory can be used to explain users’ addiction to short videos. The Social Shaping of Technology theory adopts a constructivist approach to study the social formation process of technology. It suggests that technology is shaped by social factors and should be open to sociological investigations. It applies sociological methods to examine how social, political, economic, and cultural forces influence the formation of technology, providing a new perspective for understanding the relationship between technology and society. This theory emerged as a reflection and critique of “technological determinism” and differs from traditional perspectives that solely focus on the outcomes of technological progress. Instead, it shifts the research focus to social factors beyond the technology itself and examines the specific processes involved in the content and innovation of technology. Specifically, the key point of the Social Shaping of Technology theory is that each stage of the emergence and implementation of new technology involves choices between different technological options, and each choice is directly or indirectly influenced by society or individuals, which in turn has corresponding impacts on individuals and society (Williams and Edge, 1996). In other words, technology is not in a “black box” state. Only by opening the “black box” of technology and analyzing the socio-economic patterns in the process of technological innovation can we truly understand the origins of technology and its impacts.

The implementation of personalized recommendations for short video content relies on recommendation algorithms. However, the continuous “evolution” of recommendation algorithms is not solely the work of engineers but gradually achieved under the driving force of user feedback. As the level of internet application services continues to improve, users have more specific and precise demands for internet content. Personalized recommendation algorithms have emerged and developed under the impetus of user behavior data accumulation. However, when facing addiction to short videos, it is not appropriate to simply attribute the responsibility to recommendation algorithms or short video platforms. Instead, a deeper reflection on technological products such as short videos should be conducted from the perspectives of social development and user needs. For example, why do different users have different attitudes toward short videos? If the shaping of technology by society or individuals is ignored and the impact of short videos on individuals and society is analyzed unilaterally, the measures taken to prevent short video addiction may lag or be ineffective.

## 5 Recommendations for addressing short video addiction

Short video addiction is a new hot topic in current research. How can we address the negative impacts of short video addiction? Specific recommendations can be made from both theoretical research and practical applications.

### 5.1 Research outlook

At the theoretical research level, there are three areas that deserve future attention. Firstly, research methods and ideas need to be further developed and enhanced. Different research approaches can address the methodological needs of a particular scientific problem from various research perspectives. As a result, these approaches have the potential to uncover multiple facets of the problem. In summary, the majority of existing studies on short video addiction have relied on research data obtained through participants’ questionnaire reports (e.g., Feng et al., 2022), and the specificity of short video addiction was not fully considered. Some scholars have utilized qualitative research to propose a targeted measurement method for short video addiction (Luo, 2022). However, this approach also encompasses users’ engagement with other content on short video platforms, such as collecting gold coins. This expansion has extended beyond the realm of short videos, resembling more of an addiction assessment for a short video platform. Indeed, a similar situation has previously occurred when certain scholars developed measurement schemes for Internet addiction (Lortie and Guitton, 2013). Future research could seek to enhance the exploration of short video addiction through the following approaches: (1) Develop a brief video addiction scale through interviews or expert assessment (refer to Hsu et al., 2015) to evaluate whether users are addicted to short videos; (2) Conduct behavioral experiments, including randomized controlled trials and delay discounting tasks, to investigate users’ choice preferences, decision-making ability, self-regulation strategies, and other aspects under different conditions. This approach will help in understanding the behavioral characteristics and basic needs associated with short video addiction; (3) Utilize neuroimaging techniques (refer to Su et al., 2021), such as functional magnetic resonance imaging and electroencephalography, to observe the brain activity of individuals while they are watching short videos. By analyzing the neural mechanisms underlying user attention, emotion, and reward systems, it is beneficial to uncover the neural basis of addiction to short videos.

Secondly, there is a need to deepen the understanding of the mechanisms behind short video addiction. The key to addressing the issue lies in gaining a profound insight into its nature. However, existing research primarily focuses on the harms of short video addiction while neglecting the mechanisms of addiction, resulting in a limited understanding of the problem within the academic community and the inability to propose practical and effective governance measures. As mentioned earlier, algorithm design, content services, platform control, and user experience are the four key aspects involved in short video usage and are also potential causes of addiction. The intelligent technology, diverse content, convenience of platforms, and user experience in short video services differ from traditional social media. These characteristics may intertwine with user needs and psychological traits, leading to the occurrence of short video addiction. In future research, it is necessary to not only delve into the external triggering mechanisms of short video addiction but also further explore the psychological and behavioral factors of addiction among short video users. It can be said that the analysis of external triggering mechanisms and the exploration of internal mechanisms of short video addiction will jointly contribute to clarifying the origins of the problematic behavior, thereby providing scientific guidance for the development of preventive and intervention measures. Furthermore, by raising public awareness of the internal mechanisms of short video addiction, we can enhance societal understanding and recognition of this potential risk, achieving a fundamental effect. For example, from the perspective of short videos, personalized recommendation algorithms are one of the key factors that contribute to short video addiction (Zhao, 2021). Algorithm literacy, which safeguards users’ autonomy and decision-making rights, may become an important knowledge reserve (Bakshy et al., 2015). Therefore, disseminating knowledge about algorithms and their operating principles can enhance users’ rational thinking and coping abilities, enabling them to resist the negative impacts of algorithms and achieve collective prevention of short video addiction.

Thirdly, the impact of recommendation algorithms on short video addiction should be considered. Issues such as gaming addiction and problematic use of social networking sites have been widely studied, but few scholars have explored the causes of addiction from a technological or device perspective. Short video addiction, as a newly emerging problem triggered by technology, is particularly relevant in this regard. It is well known that the development of algorithms has improved work efficiency in various industries and brought about new opportunities. However, while algorithms bring convenience to people, the risks and challenges they pose have also raised concerns in various fields. The recommendation algorithms of short video platforms are constantly evolving based on market feedback, and the specific parameters of the algorithms evolve based on changes in users’ personal information and usage behavior. The more users engage with short videos, the more the recommendation algorithm caters to their preferences, leading to deeper involvement (Zhang and Liu, 2021). Unlike general social networking platforms, the key interaction in short video usage is not between users and their social networks, but between users and the so-called algorithm itself (Bhandari and Bimo, 2020). This suggests that there may be a closed loop between short video usage and algorithm optimization, and if this effect continues, it may lead to more users becoming addicted and have a greater social impact. Therefore, simply treating the technological support behind short video services as a black box is not a long-term solution, as neglecting personalized algorithms in studying the impact of short videos on users may not truly address the core issue. Considering this, it is worth considering the “supply-side” perspective: if platforms blindly pursue rich user profiles and precise content recommendations, it will only lead to more users becoming addicted.

### 5.2 Practical measures

On the practical application level, short videos are a double-edged sword for users, especially minors. When directed toward correct, meaningful, and valuable content, short videos can provide knowledge and education. However, negative content in short videos can easily lead users, especially minors, to become addicted to shallow pleasures, which can harm their physical and mental health and even affect the formation of their correct values. It is evident that the problem of short video addiction is a complex social issue that requires the joint efforts of society. Therefore, we can clarify the obligations and responsibilities of relevant parties such as short video producers, platforms, and regulators in preventing short video addiction through legislation.

Firstly, from the perspective of short video producers, influencers are the backbone of maintaining the popularity of short video platforms. The “Douyin Research Report” shows that influencers who are similar to the respondents account for only 4.7% of the total number of platform users, but they have a staggering 97.7% of the total number of fans, and the top 2.7% of their videos attract more than 80% of the platform users’ attention and participation (Douyin Research Report, 2018). In practice, influencers or other content creators often adhere to the rule of “traffic first.” While they may gain immediate benefits, in the long run, it can deplete their vitality. After experiencing explosive growth, short video platforms must inevitably face long-term production and consumption (Peng, 2019). Attracting users relies on high-quality content. Therefore, as the source of content, short video producers should improve their own literacy, adhere to the principle of “content first,” and produce more high-quality short videos that guide users correctly, are rich in content, and provide educational and entertaining value.

Secondly, from the perspective of platforms, the issue of addiction reflects the commercial nature of short video platforms, which prioritize profitability. The power wielded by these platforms poses a potential threat to public communication, ideological security, and social aesthetics (Zhang and Liang, 2023). Therefore, platforms should reflect on the entire process of their commercial operations and fulfill their responsibilities as entities, avoiding the control of public resources by commercial capital. In fact, it is not unreasonable to expect short video platforms to take on more regulatory responsibility for the frequent chaos in the field of short videos. Many large platforms have a large user base and a significant impact on minors. This requires platforms to first refrain from using algorithmic recommendation services to induce minors to become addicted to the internet, and it is strictly prohibited to promote violent, pornographic, or other inappropriate videos to them. Then, short video platforms should upgrade their “anti-addiction systems” using identity recognition and big data analysis and improve the short video rating system to prevent harmful content that affects the physical and mental health of users, especially minors, from being published on the platform. As a result, platforms must accurately classify, manage, and rigorously audit short videos to ensure that they are more suitable for users within specific age groups. For instance, platforms can enhance and strengthen the short video anti-addiction system through technical improvements such as interface design, content distribution, content push and time management. These measures will contribute to a more equitable balance between commercial interests and social responsibility. Therefore, in addition to legal obligations, platforms should also take on more social and moral responsibilities. In this process, short video platforms need to consider various issues, such as the potential increase in costs. However, at the same time, platforms will gain a good reputation, and society will recognize their business practices, leading to a corresponding market share. It is evident that assuming more responsibility can help short video platforms enter a virtuous cycle of development.

Thirdly, from the perspective of regulators, an efficient regulatory system is crucial to addressing the problem of short video addiction. Since regulation plays a major role in China, and the impact of government regulation on the occurrence and development of short video addiction can be hardly ignored, all sectors of society hope to see a resolution to the issue of short video addiction. However, relying solely on the efforts of platforms is not sufficient; it also requires the government to establish a scientific and efficient regulatory system. In fact, the Chinese government has taken a series of measures to solve the problem of short video addiction. In 2023, the special campaign, named “Qinglang Operation and Rectify the Problem of Poor Guidance of Short Video Information,” was launched by Chinese government, focusing on three prominent problems of spreading false information, displaying improper behavior and spreading wrong ideas. Among them, short videos displaying improper behavior, such as “pornographic edge” behavior, creating vulgar people, malicious marketing of Internet celebrities and displaying high-risk behaviors, are most likely to cause short video addiction and become the focus of regulation. In the future, China should continue to build upon the existing measures and further improve the normalized short video supervision measures to promote the standardized development of the short video industry. First of all, China should strengthen legislation and real-name system management, especially to constrain the identity of minors, and strive to urge platforms to standardize content review and functional operation, and promote the fundamental transformation from “management” to “governance.” Therefore, the government could impose restrictions on the content released by short video platforms, their operational mechanisms, user access and online time in order to ensure that platforms are compliant with regulations and operate legally. Then, regulatory authorities need to strengthen supervision and implement comprehensive regulation through measures such as online review and application control. Specifically, we can establish the position of the Cyberspace Administration as the primary regulator, clarifying its responsibilities and division of labor with the Public Security Bureau, Education Bureau, Culture Bureau, Health Commission, and Market Supervision Administration. This includes information sharing and coordinated law enforcement to improve regulatory efficiency. Meanwhile, China should strengthen regulatory oversight and implement a rigorous system of accountability at all levels of government to effectively control the issue of short video addiction. This will be essential in achieving successful prevention and control measures. Finally, we can fully leverage the proactive role of industry organizations in the internet sector to fulfill their regulatory functions, serving as an effective complement to government oversight. For instance, with regards to capital supervision, China should allocate resources toward the construction of public platform network services. It is important to establish a non-exploitative platform that prioritizes user service and ensures the protection of the legitimate rights and interests of short video users, particularly minors. Only in this way can we maximize the positive effects of multi-party governance, promote the healthy development of short video platforms, and make them a cultural home for the masses.

## Author contributions

ML: Conceptualization, Formal analysis, Investigation, Writing – original draft, Writing – review & editing.

## References

[ref1] Alter A. (2018). Irresistible: the rise of addictive technology and the business of keeping us hooked. (J. Lu, Trans.). Beijing: Machinery Industry Press.

[ref2] Bakshy E. Messing S. Adamic L. A. (2015). Exposure to ideologically diverse news and opinion on Facebook. Science 348, 1130–1132. doi: 10.1126/science.aaa1160, PMID: 25953820

[ref3] Bhandari A. Bimo S. (2020). TikTok and the “algorithmized self”: a new model of online interaction. AoIR Sel. Pap. Internet Res. doi: 10.5210/spir.v2020i0.11172

[ref4] Carta I. Chen C. H. Schott A. L. Dorizan S. Khodakhah K. (2019). Cerebellar modulation of the reward circuitry and social behavior. Science 363:eaav0581. doi: 10.1126/science.aav0581 30655412 PMC6711161

[ref5] Casey B. J. (2013). The teenage brain: an overview. Curr. Dir. Psychol. Sci. 22, 80–81. doi: 10.1177/0963721413486971 PMC4182916 25284961

[ref6] China Health Education Center (2018). The core messages and interpretation of health education for Chinese adolescents (2018 edition). Health Guide 6:46.

[ref7] China Internet Network Information Center. (2023a). Statistical report on internet development in China, August 28, 2023. Available at: https://www.cnnic.net.cn/n4/2023/0828/c88-10829.html

[ref8] China Internet Network Information Center. (2023b). Statistical survey on internet development in China, December 25, 2023. Available at: https://www.cnnic.cn/n4/2023/1225/c116-10908.html

[ref9] Chou T. J. Ting C. C. (2003). The role of flow experience in cyber-game addiction. Cyber Psychol. Behav. 6, 663–675. doi: 10.1089/109493103322725469, PMID: 14756934

[ref10] Cinelli M. de Francisci Morales G. Galeazzi A. Quattrociocchi W. Starnini M. (2021). The echo chamber effect on social media. Proc. Natl. Acad. Sci. 118:e2023301118. doi: 10.1073/pnas.2023301118, PMID: 33622786 PMC7936330

[ref11] Douyin Research Report. (2018). Miao Zhen & Haimayun. Available at: http://www.199it.com/archives/769843.html (Accessed September 11, 2018).

[ref14] Feng Y. Li L. Zhao A. (2022). A cognitive-emotional model from mobile short-form video addiction to intermittent discontinuance: the moderating role of neutralization. Int. J. Hum. Comput. Interact. 40, 1505–1517. doi: 10.1080/10447318.2022.2147714

[ref15] Geschke D. Lorenz J. Holtz P. (2019). The triple-filter bubble: using agent-based modelling to test a metatheoretical framework for the emergence of filter bubbles and echo chambers. Br. J. Soc. Psychol. 58, 129–149. doi: 10.1111/bjso.12286, PMID: 30311947 PMC6585863

[ref16] Griffiths M. (2000). Internet addiction-time to be taken seriously? Addict. Res. 8, 413–418. doi: 10.3109/16066350009005587

[ref17] He Q. Zhou H. (2021). Investigating the phenomenon of short video addiction among adolescents. Contemp. Commun. 2021, 102–104.

[ref9001] Hsu W. Y. Lin S. S. Chang S. M. Tseng Y. H. Chiu N. Y. (2015). Examining the diagnostic criteria for Internet addiction: Expert validation. Journal of the Formosan Medical Association 114, 504–508. 24787664 10.1016/j.jfma.2014.03.010

[ref18] Jia Y. Liu T. Y. Yang Y. (2023). Trapped in the smartphone: intergenerational relationships and internet addiction among older adults. J. Bimonthly 10, 31–44.

[ref19] Kircaburun K. Griffiths M. D. (2018). Instagram addiction and the big five of personality: the mediating role of self-liking. J. Behav. Addict. 7, 158–170. doi: 10.1556/2006.7.2018.15, PMID: 29461086 PMC6035031

[ref20] Koob G. F. Volkow N. D. (2010). Neurocircuitry of addiction. Neuropsychopharmacology 35, 217–238. doi: 10.1038/npp.2009.110, PMID: 19710631 PMC2805560

[ref21] Lee Z. W. Cheung C. M. Chan T. K. (2014). Explaining the development of the excessive use of massively multiplayer online games: a positive-negative reinforcement perspective. 2014 47th Hawaii International Conference on System Sciences (pp. 668–677). IEEE.

[ref22] Leung L. (2020). Exploring the relationship between smartphone activities, flow experience, and boredom in free time. Comput. Hum. Behav. 103, 130–139. doi: 10.1016/j.chb.2019.09.030

[ref23] Leung L. Chen C. (2021). A review of media addiction research from 1991 to 2016. Soc. Sci. Comput. Rev. 39, 648–665. doi: 10.1177/0894439318791770

[ref24] Li L. (2018). Analysis of the “Pseudo-neutrality” of online intelligent recommendation algorithms. Modern Commun. 8:84.

[ref25] Li X. Qin H. X. Zeng M. H. He Y. X. Ma M. Z. (2021). Relationship between symptoms of short video addiction and personality traits among college students. Chinese J. Mental Health 35, 925–928.

[ref26] Liang Q. (2023). Guide bias and bidirectional inversion: a study of short video addiction among rural left-behind children. New Theory Tianfu 4, 118–131.

[ref27] Liu C. C. Chang I. C. (2016). Model of online game addiction: the role of computer-mediated communication motives. Telematics Inform. 33, 904–915. doi: 10.1016/j.tele.2016.02.002

[ref28] Lortie C. L. Guitton M. J. (2013). Internet addiction assessment tools: dimensional structure and methodological status. Addiction 108, 1207–1216. doi: 10.1111/add.12202, PMID: 23651255

[ref29] Luo G. F. (2022). Development and application of a scale for college students’ addiction to short videos. Hubei: Changjiang University.

[ref30] Masciantonio A. Bourguignon D. Bouchat P. Balty M. Rimé B. (2021). Don’t put all social network sites in one basket: Facebook, Instagram, Twitter, TikTok, and their relations with well-being during the COVID-19 pandemic. PLoS One 16:e0248384. doi: 10.1371/journal.pone.0248384, PMID: 33705462 PMC7951844

[ref31] Media Review. (2023). Research report on the development of internet audiovisual in China 2023, sarft.net, March 29, 2023. Available at: https://www.sarft.net/a/214357.aspx

[ref32] Meng K. S. Leung L. (2021). Factors influencing TikTok engagement behaviors in China: an examination of gratifications sought, narcissism, and the big five personality traits. Telecommun. Policy 45:102172. doi: 10.1016/j.telpol.2021.102172

[ref33] Michailidis L. Balaguer-Ballester E. He X. (2018). Flow and immersion in video games: the aftermath of a conceptual challenge. Front. Psychol. 9:393107. doi: 10.3389/fpsyg.2018.01682 PMC6134042 30233477

[ref34] Nong W. He Z. Ye J. H. Wu Y. F. Wu Y. T. Ye J. N.. (2023). The relationship between short video flow, addiction, serendipity, and achievement motivation among Chinese vocational school students: the post-epidemic era context. Healthcare 11:462. doi: 10.3390/healthcare11040462, PMID: 36832995 PMC9957412

[ref35] Peng L. (2019). Short videos: “transgenic” and re-cultivation of video productivity. Journalism:36.

[ref9003] Ponnusamy S. Iranmanesh M. Foroughi B. Hyun S. S. (2020). Drivers and outcomes of Instagram Addiction: Psychological well-being as moderator. Computers in human behavior 107:106294.

[ref36] Qin Y. Omar B. Musetti A. (2022). The addiction behavior of short-form video app TikTok: the information quality and system quality perspective. Front. Psychol. 13:932805. doi: 10.3389/fpsyg.2022.932805, PMID: 36148123 PMC9486470

[ref37] Ryan T. Chester A. Reece J. Xenos S. (2014). The uses and abuses of Facebook: a review of Facebook addiction. J. Behav. Addict. 3, 133–148. doi: 10.1556/JBA.3.2014.016, PMID: 25317337 PMC4189307

[ref38] Sanz-Blas S. Buzova D. Miquel-Romero M. J. (2019). From Instagram overuse to instastress and emotional fatigue: the mediation of addiction. Spanish J. Market. ESIC 23, 143–161. doi: 10.1108/SJME-12-2018-0059

[ref39] Saunders C. Over D. E. (2009). “In two minds about rationality” in In two minds: dual processes and beyond. eds. Evans J. S. B. T. Frankish K. (New York: Oxford), 317–334.

[ref40] Seo D. Ray S. (2019). Habit and addiction in the use of social networking sites: their nature, antecedents, and consequences. Comput. Hum. Behav. 99, 109–125. doi: 10.1016/j.chb.2019.05.018

[ref9002] Shiffrin R. M. Schneider W. (1977). Controlled and automatic human information processing: II. Perceptual learning, automatic attending and a general theory. Psychological review, 84:127.

[ref41] Southern Metropolis Daily. (2009). Ministry of Health denies concept of “internet addiction,” advises against making minors quit the internet, China News Network. Available at: https://www.chinanews.com.cn/it/it-itxw/news/2009/11-05/1948561.shtml (Accessed November 5, 2009).

[ref42] Strongman K. T. (2006). The psychology of emotion from everyday life to theory (the 5th edition). Wang Li, Trans. Beijing: China Light Industry Press 7–18, 69–92.

[ref43] Su C. Zhou H. Gong L. Teng B. Geng F. Hu Y. (2021). Viewing personalized video clips recommended by TikTok activates default mode network and ventral tegmental area. NeuroImage 237:118136. doi: 10.1016/j.neuroimage.2021.118136, PMID: 33951514

[ref44] Tian X. (2023). Research on short video user behavior from the perspective of experience value. Jilin: Jilin University.

[ref45] Tian X. Bi X. Chen H. (2023). How short-form video features influence addiction behavior? Empirical research from the opponent process theory perspective. Inf. Technol. People 36, 387–408. doi: 10.1108/ITP-04-2020-0186

[ref46] van Rooij A. Prause N. (2014). A critical review of “internet addiction” criteria with suggestions for the future. J. Behav. Addict. 3, 203–213. doi: 10.1556/JBA.3.2014.4.1, PMID: 25592305 PMC4291825

[ref47] Volkow N. D. Michaelides M. Baler R. (2019). The neuroscience of drug reward and addiction. Physiol. Rev. 99, 2115–2140. doi: 10.1152/physrev.00014.2018, PMID: 31507244 PMC6890985

[ref48] Wang X. (2021). Analysis of the causes, potential risks and countermeasures of the phenomenon of short video fever in college students. Ideol. Theor. Educ. 1, 93–97.

[ref49] Wang J. Sheng J. Wang H. (2019). The association between mobile game addiction and depression, social anxiety, and loneliness. Front. Public Health 7:476493. doi: 10.3389/fpubh.2019.00247 PMC6743417 31552213

[ref50] Wilbur S. (1984). A look at human communication (Translated by Chen Liang et al.): Xinhua Publishing House, 114.

[ref51] Williams R. Edge D. (1996). The social shaping of technology. Res. Policy 25, 865–899. doi: 10.1016/0048-7333(96)00885-2

[ref52] Xie X. Du Y. (2023). An empirical study on the influence of socio-technical factors on short video addiction. Moderat. Effect Recomm. Algorith. 1, 72–75.

[ref53] Yan W. Liu S. Zhang R. Xu P. (2021). Susceptibility of compulsivity traits and the neural basis of prefrontal-Antireward system in drug addiction behavior. Adv. Psychol. Sci. 29, 1345–1357. doi: 10.3724/SP.J.1042.2021.01345

[ref54] Yang S. Lu Y. Wang B. Zhao L. (2014). The benefits and dangers of flow experience in high school students’ internet usage: the role of parental support. Comput. Hum. Behav. 41, 504–513. doi: 10.1016/j.chb.2014.09.039

[ref55] Yu G. Jiao J. Zhang X. (2015). The origin, theory, and operational key of “platform media”. J. Renmin Univ. China 6:121.

[ref56] Zea Q. Jung H. (2019). Learning and sharing creative skills with short videos: A case study of user behavior in Tiktok and Bilibili. Manchester, UK: International Association of Societies of Design Research (IASDR), Design Revolution.

[ref57] Zhang G. Liang X. (2023). Control and capture: the role of platforms in the phenomenon of short video addiction. Modern Commun. 11, 81–84.

[ref58] Zhang M. Liu Y. (2021). A commentary of TikTok recommendation algorithms in MIT technology review 2021. Fundam. Res. 1, 846–847. doi: 10.1016/j.fmre.2021.11.015

[ref59] Zhang X. Wu Y. Liu S. (2019). Exploring short-form video application addiction: socio-technical and attachment perspectives. Telematics Inform. 42:101243. doi: 10.1016/j.tele.2019.101243

[ref60] Zhao Z. (2021). Analysis on the “Douyin (Tiktok) mania” phenomenon based on recommendation algorithms. E3S Web Conf. 235:03029.

